# Intravenous Administration of Bone Marrow-Derived Mesenchymal Stem Cells Induces a Switch from Classical to Atypical Symptoms in Experimental Autoimmune Encephalomyelitis

**DOI:** 10.1155/2015/140170

**Published:** 2015-03-09

**Authors:** Mónica Kurte, Javiera Bravo-Alegría, Alexander Torres, Vania Carrasco, Cristina Ibáñez, Ana María Vega-Letter, Catalina Fernández-O'Ryan, Carlos E. Irarrázabal, Fernando E. Figueroa, Rodrigo A. Fuentealba, Claudia Riedel, Flavio Carrión

**Affiliations:** ^1^Laboratory of Cellular and Molecular Immunology, Faculty of Medicine, University of Los Andes, 750000 Santiago, Chile; ^2^Laboratory of Integrative and Molecular Physiology, Faculty of Medicine, University of Los Andes, 750000 Santiago, Chile; ^3^Department of Biological Sciences, Faculty of Biological Sciences, Andrés Bello National University, 8370146 Santiago, Chile

## Abstract

Potent immunosuppressive and regenerative properties of mesenchymal stem cells (MSCs) position them as a novel therapy for autoimmune diseases. This research examines the therapeutic effect of MSCs administration at different disease stages in experimental autoimmune encephalomyelitis (EAE). Classical and atypical scores of EAE, associated with Th1 and Th17 response, respectively, and also Treg lymphocytes, were evaluated. MSCs administration at the onset (EAE+MSC_onset_) induced an important amelioration of the clinical signs and less lasting effect at the peak of EAE (EAE+MSC_peak_). No effect was observed when MSCs were applied after EAE stabilization (EAE+MSC_late_). Surprisingly, EAE atypical signs were detected in EAE+MSC_peak_ and EAE+MSC_late_ mice. However, no correlation was found in Th17/Th1 ratio. Interestingly, regardless of time administration, MSCs significantly reduced IL-6 and also T-bet, ROR*γ*T, and Foxp3 mRNA levels in brain samples of EAE mice. The downregulation of IL-6 could restore the well-functioning of the blood-brain barrier of EAE mice, correlated with a decreased number of brain infiltrating leukocytes. These results suggest that the inflammatory status is important to be considered for administering MSCs in autoimmune pathologies, leading to a further research to clarify the effect of MSCs for multiple sclerosis.

## 1. Introduction

Mesenchymal stem cells (MSCs) are adherent, undifferentiated, pluripotent, and nonhematopoietic progenitor cells principally located in bone marrow and adipose tissue, among others [1, 2].* In vitro* expanded MSCs have the potential to differentiate into mesodermal lineages including osteoblasts, chondrocytes, and adipocytes. Furthermore, mounting evidence demonstrates usefulness for the use of MSCs in tissue repair [3, 4]. Currently, MSCs are also known for their ability to regulate the immune system. The first reports in 2002-2003 showed that MSCs are able to inhibit T cell proliferation by cell cycle arrest in G0-G1 phase [5–7]. Today, it is well accepted that MSCs have important immunosuppressive properties over the entire immune system, mainly exerting their effects on T, B, NK, and dendritic cells [8, 9].

This immunomodulatory capacity of MSCs has opened new therapeutic prospects in the management of proinflammatory and autoimmune pathologies [10–12]. The therapeutic potential of MSCs has been demonstrated in a variety of autoimmune disease models including graft-*versus*-host disease (GVDH) [13], experimental autoimmune encephalomyelitis (EAE) [14, 15], collagen-induced arthritis (CIA) [16, 17], MRL/lpr lupus-prone mice [18], and murine model of autoimmune myocarditis [19]. However, treatments with MSCs in these experimental models did not always translate into a decrease of the disease phenotype as expected. In some cases, no beneficial effects were observed during the course of the disease, whereas others demonstrated an exacerbation of clinical symptoms. Zappia et al. showed that MSCs administration was able to substantially reduce the clinical score of EAE animals but only when MSC injection was carried out before the onset [14]. Likewise, Djouad et al. observed that treatment with MSCs conferred no benefit on arthritic animals; and they showed exacerbation of symptoms when MSCs were infused at later stage of the disease [16]. These authors suggested that MSCs were associated with an increase of Th1 response and established that timing of MSCs application is important for MSC-mediated immunosuppression* in vivo*.

In our previous studies we showed that MSCs inhibit the activation and differentiation of T helper lymphocytes type 1 (Th1) [20]. Unexpectedly, we also observed that MSCs were able to promote the expansion of Th17 lymphocytes when cocultured with previously activated CD4+ T cells. The opposite effect was observed in Th1 response [20]. Likewise, Guo et al. reported that human MSCs were able to induce Th17 cells, but not Th1 in cocultures of MSCs with naive CD4+ T cells [21]. These data suggest that the MSCs have the capability to generate an imbalance on Th1/Th17 ratio and therefore to change the phenotype of the immune response* in vivo.*

Multiple sclerosis (MS) is a chronic inflammatory disease characterized by the infiltration of immune system cells to the central nervous system (CNS), causing damage to neuronal axons by loss of myelin [22]. Several studies, using the MS animal model experimental autoimmune encephalomyelitis (EAE), clearly showed that recruitment of T helper lymphocytes to the CNS is essential for the initiation of the disease [23]. Initially, Th1 lymphocytes were attributed as being primarily responsible for the demyelination of neurons [24, 25]. However Korn et al. showed that Th17 lymphocytes would be important both at the beginning and at the peak of the disease and possibly Treg lymphocytes at later stages [26]. Moreover, it has been reported that MOG (myelin oligodendrocyte glycoprotein) specific, highly pathogenic Th1 lymphocytes are necessary to allow Th17 entry into the CNS [27]; and it has been suggested that the proportion between Th1 and Th17 determines the site where inflammation occurs in the CNS [28]. Recent findings suggest that Th1 and Th17 lymphocytes generate different clinical signs of EAE. Th1 lymphocytes preferentially induce classic signs of the disease, defined as progressive paralysis of the limbs, from tail to head. On the other hand, Th17 lymphocytes induce atypical symptoms of EAE, characterized by an inability to control coordinate movements and ataxia [28, 29]. Despite divergent Th1- and Th17-induced pathologies, the* in vivo* interplay between these pathogenic lymphocyte subtypes appears to be highly complex. Although additional research is required to clearly define the individual contribution of Th1 and Th17 cells in MS, the existence of a distinct phenotype for MOG-responsive T helper lymphocytes makes the EAE model ideal to study the switch from Th1 to Th17 pathology induced by MSCs.

The aim of this study was twofold. Firstly, it attempts to clarify the therapeutic effect of MSCs injections associated with different degrees of inflammatory conditions using the EAE model. Secondly, because we have previously demonstrated that MSCs are able to promote* in vitro* Th17 lymphocytes in a proinflammatory context [20], we evaluated if MSCs induce the appearance of atypical signs of EAE associated with an increase on Th17 lymphocytes. Our data indicate that early treatment with MSCs is highly effective in reducing the clinical score of EAE. We propose that the increase in plasma cytokine levels licenses the MSCs* in vivo*, which would increase their immunosuppressive capacity. Conversely, despite decreasing inflammation, the late administration of MSCs when plasma cytokine levels are low was not as effective in reducing the clinical score of EAE as the early treatment and even induced the appearance of atypical signs of EAE, but no association with increased Th17 cell response was found. Finally, we postulate that the effect of MSCs administration, independent of the time of administration, was able to diminish the lymphocyte infiltration into the brain, probably due to a restoration of the blood-brain barrier (BBB) consequence of the decrease level of IL-6.

## 2. Methods

### 2.1. Animals

Females C57BL/6 mice, 8–14 weeks old, were purchased from the Central Animal Facility, Faculty of Medicine, University of Chile. Animals were housed under standard laboratory conditions and with food and water* ad libitum.* Experimental procedures and protocols were performed according to the US National Institutes of Health Guide for the Care and Use of Laboratory Animals (NIH publication number 85-23, revised in 1996) and were approved by the Institutional Animal Care and Use Committee of the Universidad de los Andes and the Fondecyt Bioethics Advisory Committee in Chile.

### 2.2. MSCs Isolation and Culture

Murine bone marrow-derived stem cells (MSCs) were obtained from 8–10-week-old female C57BL/6 mice, as previously described [20]. Bone marrow stromal cells were collected by flushing femurs and tibias with sterile phosphate-buffered saline (PBS) and washing by centrifugation at 350 ×g for 10 min at room temperature. After centrifugation, cells were plated in 75 cm^2^ tissue-culture flasks at density of 2 × 10^6^/cm^2^ in complete alpha Modified Eagle's Medium (*α*–MEM, Gibco, Auckland, New Zealand), containing 15% heat-inactivated MSC-qualified fetal bovine serum (FBS, Gibco), 100 U/mL penicillin, and 100 ug/mL streptomycin (Gibco) at 37°C in a 5% CO_2_ atmosphere. Nonadherent cells were removed after 72 hrs and the remaining cells were cultured until reaching 90% confluence and the medium was replenished every 3 days. After 2 passages, cells were isolated by negative selection using anti-CD45 microbeads (Miltenyi, Auburn, CA) according to the manufacturer's protocol. CD45 negative cells were cultured at 10.000 cells/cm^2^ in complete *α*–MEM medium at 37°C and 5% CO_2_. After 2-3 passages, adherent cells were characterized for adipogenic, chondrogenic, and osteogenic differentiation as previously described [20]. Phenotypic characterization for hematopoietic and mesenchymal cell antigens and immunosuppressive capacity was performed by flow cytometry.

### 2.3. Flow Cytometry Analysis

MSCs phenotype was confirmed by flow cytometry based on the positivity for CD29, CD44, Sca-1, and CD90 and the absence of CD45 and CD34 antigens (all antibodies were purchased from BD Biosciences, conjugated to FITC or PE). Surface staining was performed following standard protocol. The samples were acquired in a Beckman Coulter XL flow cytometer. Data were analyzed using FCS Express 4 Plus Research Edition software.

### 2.4. Suppression of Splenocyte Proliferation

Splenocytes were isolated from C57BL/6 mice using a 70 um cell strainer in PBS buffer and stained with CFSE according to the manufacturer's instructions. CFSE-splenocytes were cultured in the presence or absence of MSCs in a 1 : 10 ratio (MSCs : splenocytes) in a complete RPMI medium, 10% FBS, 2 mM L-glutamine, 50 *μ*M *β*-mercaptoethanol, 100 U/mL penicillin, and 100 *μ*g/mL streptomycin (Gibco) at 37°C in a 5% CO_2_ atmosphere. Cells were stimulated by adding Concanavalin A (3 *μ*g/mL) and cocultures were grown for 5 days. Nonadherent cells were then harvested and surface-stained using PE-Cy5-CD4 antibody. Samples were analyzed by flow cytometry as previously described.

### 2.5. EAE Induction and MSCs Administration

Female C57BL/6 mice, 10 to 14 weeks old, were injected subcutaneously (s.c.) in the flank with 50 *μ*g of MOG_35–55_ peptide (LifeTein LLC, USA), emulsified in complete Freund's adjuvant (Difco Laboratories, Detroit, MI), and supplemented with heat-inactivated* Mycobacterium tuberculosis* H37RA (Difco Laboratories, Detroit, MI). Two and forty-eight hours later, mice received intraperitoneally (i.p.) 300 ng of Pertussis toxin (Calbiochem, La Jolla, CA). Clinical signs appeared after 10 days of EAE induction. MSCs (1 × 10^6^) diluted in PBS were intravenously (i.v.) administrated at different stages of the disease, at the onset of the disease (day 10, MSC_onset_), at the peak of the disease (day 18, MSC_peak_), or at the time of EAE stabilization (day 30, MSC_late_) (Figure 2(a)). The clinical scores and mice weights were recorded daily for 50 days. Animals were evaluated according to previously published scoring scales [29]. The classical EAE scores were assigned as follows: score 0, no disease; score 0.5, reduced tail tonus; score 1, limp tail; score 1.5, limp tail and ataxia; score 2, limp tail, ataxia, and hind limb weakness; score 2.5, at least one hind limb paralyzed/weakness; score 3, both hind limbs paralyzed/weakness; score 3.5, complete paralysis of hind limbs; score 4, paralysis until hip; score 5, dead or dying animal. The nonclassical (atypical) EAE scores were assigned as follows: score 0, no disease; score 1, head tilted slightly (ataxia, no tail paralysis); score 2, head tilted severely; score 3, inability to walk on a straight line; score 4, laying on side; score 4.5, rolling continuously unless being supported; score 5, dead or dying animal. Representative photographs of classic (Figure 2(b)) and atypical (Figure 2(c)) score are shown. For both scoring scales, the daily mean clinical scores and cumulative scores were calculated.

### 2.6. ELISA

Plasma concentrations of a broad panel of cytokines were measured by a Milliplex mouse Th17 magnetic bead panel kit (catalogue number MTH17MAG-47K, Millipore). The panel analyzed was IFN-*γ*, TNF-*α*, IL-1*β*, IL-6, IL-4, IL-10, TNF-*β*, IL-17A, IL-17F, and IL-23. Plasma samples were obtained by centrifugation (300 ×g) for 20 min at different time points after EAE induction for each experimental group. Individual plasma samples of each group at specific time were pooled and analyzed in triplicate according to the manufacturer's instructions on Luminex 200, EMD Millipore Corporation.

### 2.7. Real Time PCR

Total RNA was extracted from spinal cords and from brain left hemispheres using Trizol reagent (Invitrogen) and treated with DNAse I (Fermentas). Two *μ*g of DNAse I treated RNA was reverse-transcribed using ImProm RT and random hexamers (Promega) in 30 *μ*L total volume reaction according to the manufacturer's recommendations. PCR was performed using 2.5 *μ*L of diluted cDNA (1 : 100–1 : 500) and 10 *μ*L of primer-containing GoTaq Master Mix (Promega, 150 pmol each primer) and analyzed using Mx3000P qPCR system (Agilent Technologies). Primers used were as follows: 5′- AGC AGT GTG GAC CGT AGA TGA -3′ (*FoxP3, sense*), 5′- GGC AGG GAT TGG AGC ACT T -3′ (*FoxP3, antisense*), 5′- AGT GTA ATG TGG CCT ACT CCT -3′ (*ROR*γ*T sense*), 5′- GGC TGG TTC GGT CAA TGG G -3′ (*ROR*γ*T, antisense*), 5′- AAC CGC TTA TAT GTC CAC CCA -3′ (*T-bet, sense*), 5′- CTT GTT GTT GGT GAG CTT TAG C -3′ (*T-bet, antisense*), 5′- CGG ACA GGA TTG ACA GAT TG -3′(*18S, sense*), and 5′-CAA ATC GCT CCA CCA ACT AA -3′ (*18S, antisense*). Expression level of transcripts was normalized to 18S mRNA levels (normalizer) and to control healthy mice (control) according to the formula 2^−Δ(ΔCT)^ [30].

### 2.8. Statistical Analysis

Statistical analyses were performed using the software GraphPad Prism 5.0 (San Diego, CA, USA). Unpaired Mann-Whitney or Kruskal-Wallis tests were used to compare between the different experimental groups. Data were expressed as mean ± SEM. All *P* values <0.05 were considered statistically significant.

## 3. Results

### 3.1. MSCs Characterization

**Figure 1:**
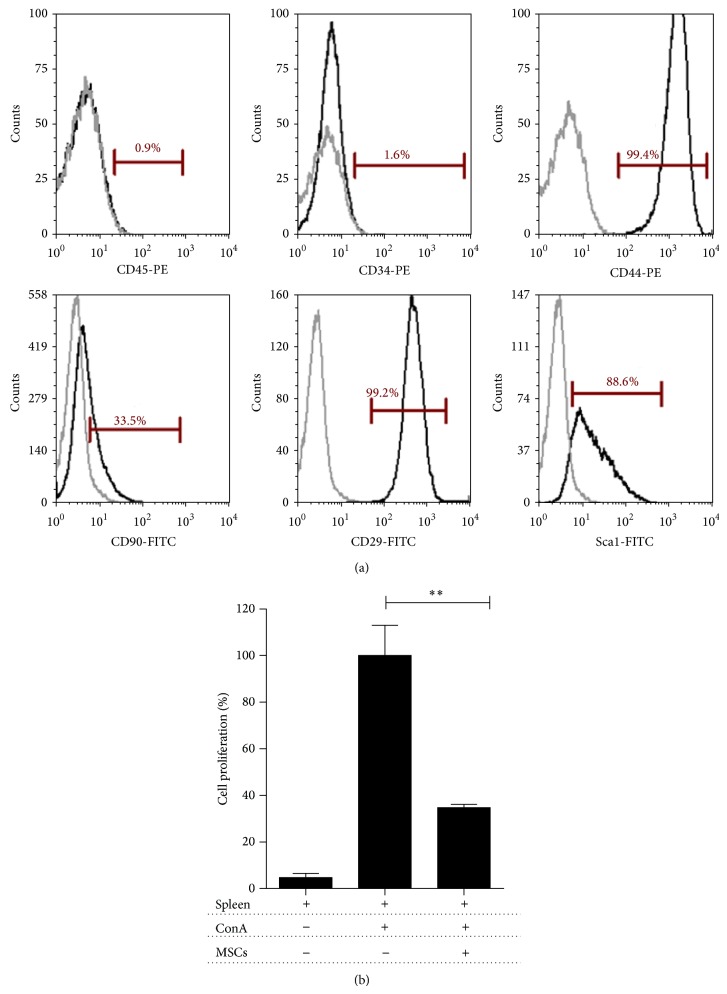
Murine MSCs characterization. (a) Phenotypic characterization of MSCs. Cells were stained for classical murine MSCs markers (CD45, CD34, CD44, CD90, CD29, and Sca-1) and evaluated by flow cytometry using a Beckman Coulter Epics XL and FCS Express V4 Plus Research Edition software. Representative histograms for each antigen are shown in black and overlapping isotype controls in grey. (b) Immunosuppressive capacity of MSCs. Splenocytes were CFSE-stained and stimulated with Concanavalin A (3 *μ*g/mL) (ConA) in the presence or absence of MSCs for 5 days. Nonstimulated cells were used as negative controls. T cell proliferation was evaluated by flow cytometry, gating on CD4+ cells. Results are shown as the mean ± SEM. *P* values were calculated using the unpaired Mann-Whitney test (^**^ *P* < 0.005), *n* = 3.

Following cell isolation, adherent cells with fibroblast-like phenotype in passage 3 were characterized following the mesenchymal stem cell minimal criteria [31]. MSCs showed high expression of the classical mesenchymal stem cells surface markers CD44, CD29, and Sca-1, milder expression of CD90 and absence of hematopoietic markers CD34 and CD45 (Figure 1(a)). The plasticity assay demonstrates that MSCs were able to differentiate into osteoblasts, chondrocyte, and adipocytes under the appropriate culture conditions as described by Carrión et al. [20]. The suppressive capacity of MSCs was determined using a proliferation assay with CFSE-splenocytes stimulated with Concanavalin A (ConA). After 5 days in coculture, MSCs inhibit the proliferation in 60% (^**^ *P* < 0.005) (Figure 1(b)).

### 3.2. MSCs Ameliorate Classical Clinical Symptoms in EAE Animals

**Figure 2:**
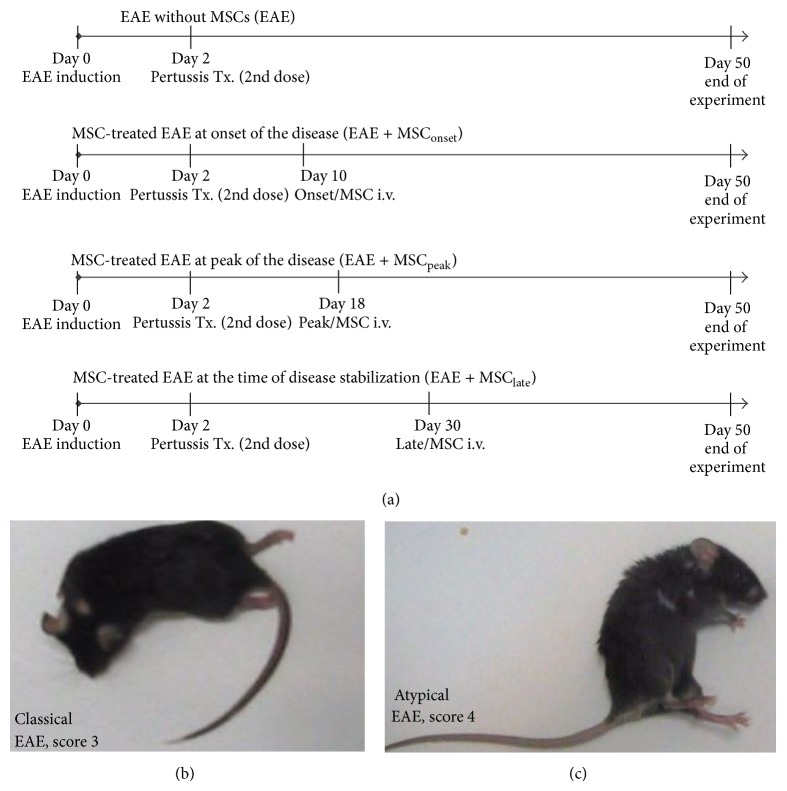
Experimental autoimmune encephalomyelitis model (EAE). (a) EAE induction. C57BL/6 mice were subcutaneously injected with 50 *μ*g of MOG_35–55_, emulsified with CFA and* Mycobacterium tuberculosis* (4 mg/mL). 2 and 48 hrs later, mice were intraperitoneally injected with Pertussis toxin (350 ng/mL). MSCs (1 × 10^6^) were intravenously administrated either at the onset (EAE + MSC_onset_), at peak of the disease (EAE + MSC_peak_), or at the time of EAE stabilization (EAE + MSC_late_). After EAE induction, animal groups were evaluated daily for both clinical score and animal weight for a total of 50 days. (b-c) Representative photographs of EAE mice manifesting the distinctive classical (b) and atypical (c) signs of the disease and their respective clinical scores are shown.

**Figure 3:**
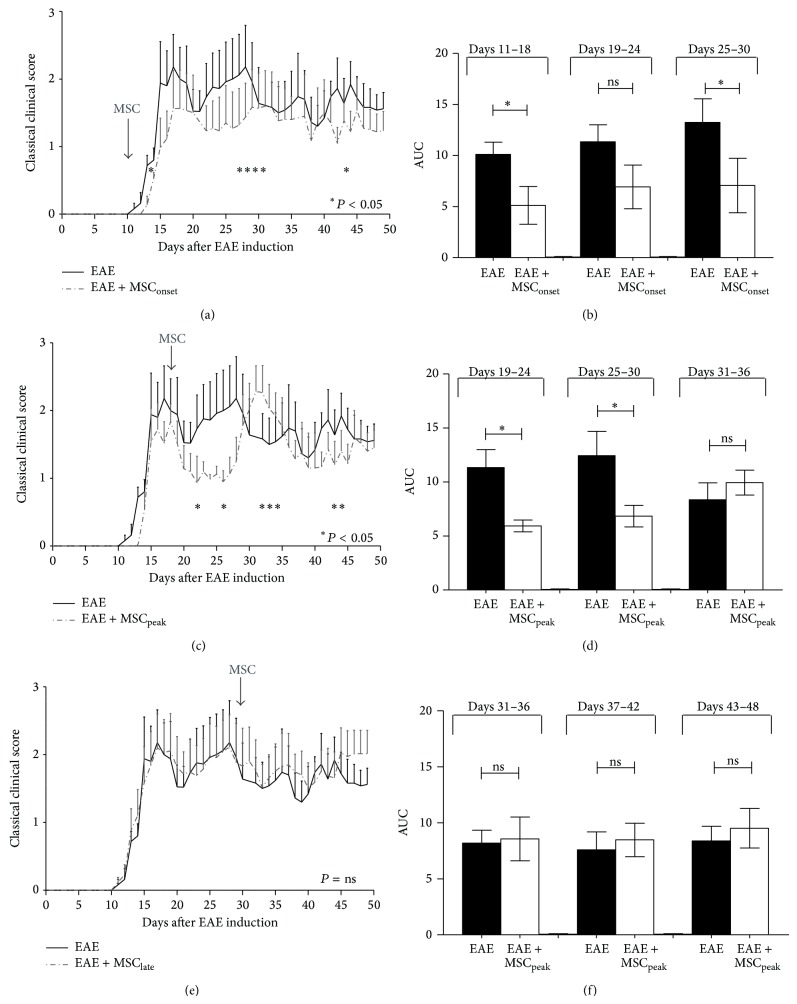
MSCs injections at the onset and at the peak of disease induce an improvement of classical EAE. C57BL/6 mice were immunized with MOG_35–55_ to induce EAE and classical score was evaluated daily. MSCs (1 × 10^6^) were intravenously injected either at (a, b) the onset of EAE symptoms (EAE + MSC_onset_), (c, d) at the peak of the disease (EAE + MSC_peak_), or (e, f) at the time of EAE stabilization (EAE + MSC_late_). Daily clinical score (a, c, e) and the area under curve (AUC) (b, d, f) parameters were evaluated after MSCs injection as described in Methods and AUC were plotted at specific time periods as indicated. Line curves and bars represent the mean ± SEM; statistical differences were calculated using Mann-Whitney test (^*^ *P* < 0.05), *n* = 7.

To elucidate the clinical effect of MSCs administration at different time points of EAE progression, MSCs were injected (1 × 10^6^ cells/mice) at the onset of the disease (day +10, MSC_onset_), at the peak of the disease (day +18, MSC_peak_), or at the time of EAE stabilization (day +30, MSC_late_) (Figure 2(a)). The classical and atypical clinical scores were evaluated for 50 days. Consistent with previous reports [14], MSCs injected at the onset of the disease induced a significant improvement of classical clinical score in EAE animals (^*^ *P* < 0.05) (Figure 3(a)). Moreover, a significant reduction of the area under curve (AUC) was detected after one (Days 11–18) and three weeks (Days 25–30) after MSC_onset_ injection, compared to the EAE group without MSCs (^*^ *P* < 0.05) (Figure 3(b)). MSCs administered at the peak of the disease induced a robust but transient improvement of clinical signs of EAE (^*^ *P* < 0.05) (Figure 3(c)). Despite this transient effect, a significant AUC was detected after one (Days 19–24) and even two weeks (Days 25–30) after MSC_peak_ injection (^*^ *P* < 0.05) (Figure 3(d)). Later administration of MSCs, at day 30, had no effect on clinical signs of classical EAE (Figure 3(e)), and we did not find differences in AUC analysis (Figure 3(f)).

### 3.3. MSCs Induced Atypical Signs of EAE

**Figure 4:**
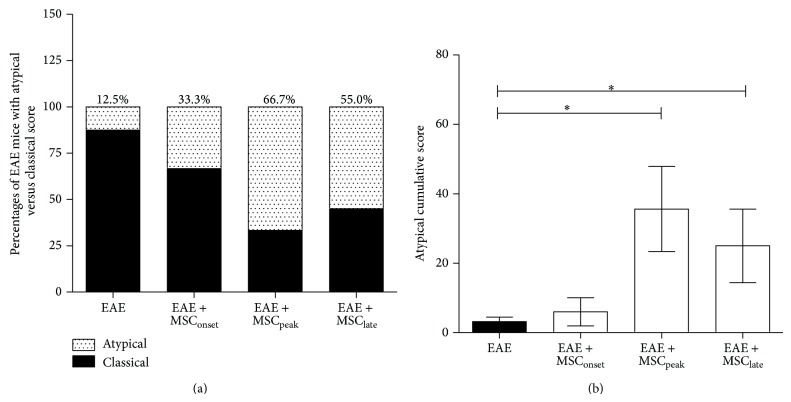
MSCs injections at the peak of EAE and at the time of EAE stabilization induce the appearance of atypical signs. EAE was induced on C57BL/6 mice by MOG_35–55_ immunization and both classical and nonclassical atypical scores were evaluated as described in Methods. (a) Incidence of classical versus atypical EAE was calculated as the percentage of EAE mice with atypical signs over EAE mice with classical phenotype at the end of the experiment. (b) Atypical cumulative scores were calculated for untreated EAE mice (EAE) and for EAE mice treated with MSCs at the onset (EAE + MSC_onset_), at the peak (EAE + MSC_peak_), and at the time of EAE stabilization (EAE + MSC_late_). Bars represent the mean ± SEM. Statistical differences were calculated using Mann-Whitney test (^*^ *P* < 0.05), *n* = 7.

Atypical EAE is characterized by unbalanced gaits or rotatory defects. The atypical condition was followed up by specific clinical score as was previously described by Domingues et al. [29]. We observed that MSCs injections were able to induce the appearance of EAE atypical signs (Figure 2(c)). An incidence of atypical EAE of 66% and 55% was observed in those animals injected at the peak (EAE + MSC_peak_) and at the time of EAE stabilization (EAE + MSC_late_), respectively. These percentages were much higher than in EAE mice without MSCs treatment (EAE, 12.5%) or in EAE mice injected at the onset with MSCs (EAE + MSC_onset_, 33.3%) (Figure 4(a)). This phenomenon was better detected, when the atypical cumulative score was compared with EAE mice treated with MSCs versus those untreated (Figure 4(b)). Higher atypical cumulative scores were observed in EAE mice treated with MSCs at the peak of the disease (EAE + MSC_peak_) and at the time of EAE stabilization (^*^ *P* < 0.05) (Figure 4(b)). We did not observe an important appearance of atypical signs when MSCs were administered at the onset or to untreated animals (EAE). These results suggest that late MSCs treatments may predispose to the appearance of atypical EAE, and therefore we wanted to analyze CNS infiltration of different types of T helper lymphocytes that are associated with these clinical signs using a molecular biology approach.

### 3.4. MSCs Diminished the Presence of T-bet, ROR*γ*T, and Foxp3 in Brains of EAE Mice

**Figure 5:**
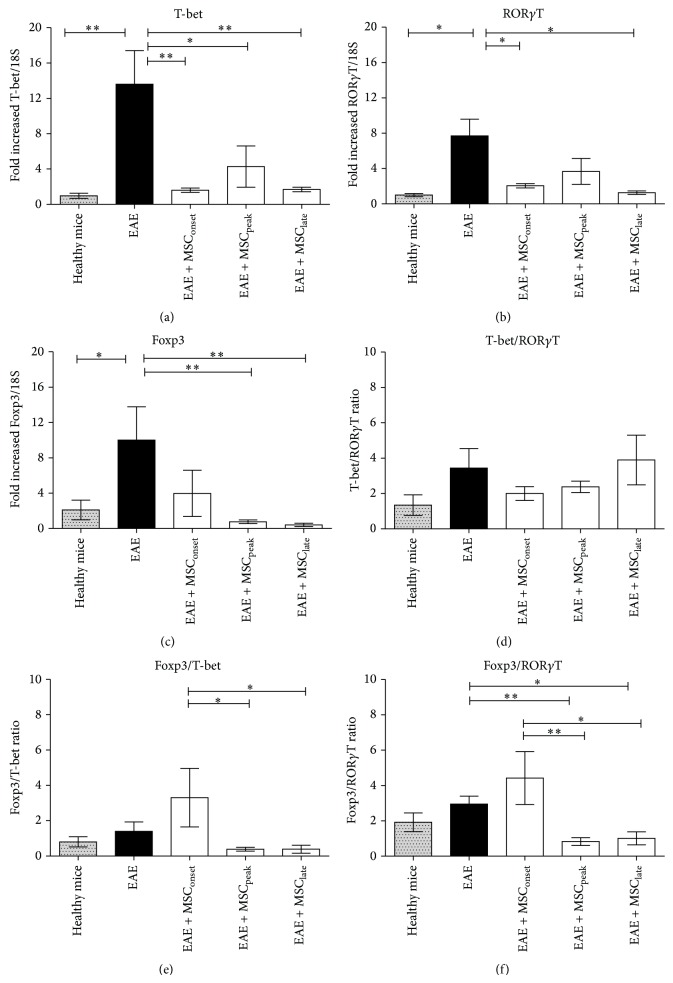
MSCs injections at different stages of EAE diminished brain T-bet, ROR*γ*T, and Foxp3 mRNA levels. Healthy C57BL/6, EAE mice, or EAE mice injected with MSCs (1 × 10^6^) at the onset (EAE + MSC_onset_), at the peak (EAE + MSC_peak_), and at the time of EAE stabilization (EAE + MSC_late_) were sacrificed after 50 days of EAE progression. Total brain RNA was extracted (Trizol) and 2 *μ*g of DNAse-treated RNA was retrotranscribed as described in Methods. Relative mRNA levels of* bona fide* transcriptions factors associated with CD4+ T helper subtypes Th1 (*T-bet*, (a)), Th17 (*ROR*γ*T*, (b)), and Treg (*Foxp3*, (c)) were determined by RT-qPCR using the 2^−ΔΔCt^ method. Individual ratios, (d) T-bet/ROR*γ*T, (e) Foxp3/T-bet, and (f) Foxp3/ROR*γ*T were also calculated. Bars in the plots represent the mean ± SEM. Statistical differences were calculated using Mann-Whitney test (^*^ *P* < 0.05, ^**^ *P* < 0.005), *n* = 7.

The presence of different subsets of T helper lymphocytes was analyzed in the CNS of these animals by qRT-PCR targeted to specific transcription factors: T-bet, ROR*γ*T, and Foxp3 associated with Th1, Th17, and Treg, respectively. Evaluation of Th1, Th17, and Treg by qRT-PCR was validated by comparative analysis between qRT-PCR and flow cytometry using* in vitro* differentiated Th1, Th17, Treg, and control cells (Supplementary Methods and Supplementary Figure 1 available online at http://dx.doi.org/10.1155/2015/140170). Fifty days after EAE induction, animals were sacrificed, brain and spinal cord samples were obtained and then total RNA was isolated as described on methods. A significant increase was detected in T-bet (^**^ *P* < 0.005), ROR*γ*T (^*^ *P* < 0.05), and Foxp3 (^*^ *P* < 0.05) in brains samples on EAE group at day 50 after EAE induction (Figure 5). The treatment with MSCs, independently of the time of administration, significantly decreased the expression level of T-bet, ROR*γ*T, and Foxp3, suggesting a lower presence of Th1, Th17, and Treg lymphocytes (^*^ *P* < 0.005, ^**^ *P* < 0.005 as indicated in Figures 5(a), 5(b), and 5(c)). The expression of T-bet, ROR*γ*T, and Foxp3 in MSCs treated groups was similar to the expression level of healthy mice, and significant differences were not found between MSCs treated groups. A slight increase of T-bet/ROR*γ*T ratio was observed in EAE + MSC_late_ and EAE group untreated with MSCs, suggesting a predominance of Th1 lymphocytes in those groups which showed the highest classical clinical score (Figure 5(d)).

Analysis of Foxp3 expression in different MSCs treated groups showed an important decrease of Foxp3 in EAE + MSC_peak_ and EAE + MSC_late_ (^**^ *P* < 0.005) but it was not so significant in EAE + MSC_onset_ (Figure 5(c)). In addition, highest Foxp3/T-bet and Foxp3/ROR*γ*T ratios were detected in brain samples of EAE + MSC_onset_ (^*^ *P* < 0.005, ^**^ *P* < 0.005 as indicated in Figures 5(e) and 5(f)), suggesting a predominance of Treg over Th1 and Th17 lymphocytes. The Foxp3/ROR*γ*T and Foxp3/T-bet ratios were <1 when MSCs were injected later (EAE + MSC_peak_ and EAE + MSC_late_), even lower than healthy mice (Figures 5(e) and 5(f)), suggesting a predominance of proinflammatory T cells versus Treg in these groups.

Except for a small detection of ROR*γ*T expression in EAE mice treated with MSCs at the peak of the disease, we were unable to detect the expression of T-bet, ROR*γ*T, or Foxp3 by qRT-PCR in spinal cord samples from all EAE groups at the end of the experiment (data not shown).

### 3.5. Evaluation of Inflammatory Profiles at Different Stages of EAE Progression

**Figure 6:**
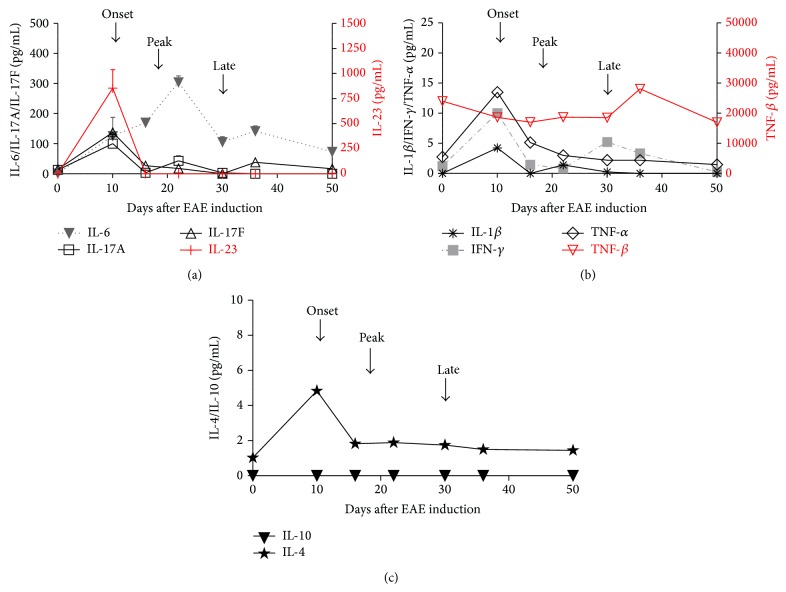
Cytokines levels in plasma samples of EAE mice at the different times of EAE progression. Blood samples were taken at different times of EAE progression, before EAE induction (day 0), at the onset (day +10), before the peak (day +16), after the peak (day +22), at the time of EAE stabilization (day +30), after EAE stabilization (day +36), and at the end of experiment (day +50). Plasma samples were isolated by centrifugation and stored at −80°C until being used. Cytokines levels were detected using Milliplex mouse Th17 magnetic bead panel on Luminex 200 instrument (Merck, Millipore) and the kinetics were plotted of (a) Th1, (b) Th17 cytokines profiles and (c) IL-4 and IL-10 of EAE mice (*n* = 7, each experiment). The samples of each experiment were pooled and analyzed independently in triplicate.

In order to evaluate the kinetics of the inflammatory response during EAE progression, the cytokine profile in plasma samples of EAE mice at different time points was assessed. Blood samples were collected either before EAE induction (day 0), at the onset (day +10), before the peak (day +16), after the peak (day +22), at the time of EAE stabilization (day +30), after EAE stabilization (day +36), or at the end of experiment (day +50). Ten different cytokines were measured using Milliplex mouse magnetic bead panel kit (IL-6, IL-1*β*, IFN-*γ*, TNF-*α*, TNF-*β*, IL-23, IL-17A, IL-17F, IL-4, and IL-10). At the onset of the disease, high levels IL-6 were detected, as well as cytokines associated with Th1 (IL-1*β*, IFN-*γ*, TNF-*α*, and TNF-*β*) (Figure 6(a)) and Th17 (IL-23, IL-17A, and IL-17F) lymphocytes (Figure 6(b)). IL-10 was not detected at any of the time points analyzed and only very low amounts of IL-4 during EAE progression were found (Figure 6(c)). In a separate experiment, IL-27 and IL-17E were also measured and at the onset of EAE high levels of IL-27 and IL-17E were found (Supplementary Figure 2). The inflammatory scenario at the peak of EAE was completely different; at this time the majority of the cytokines had already decreased to levels close to those observed in healthy mice, and only IL-6 plasma level and to a lesser extent TNF-*α* remained elevated. At the time of EAE stabilization, IL-6 levels had decreased close to those detected at the onset of EAE, and a slight increase of IFN-*γ* in respect to the peak point was observed. Levels of other cytokines remained close to those observed in healthy mice (Figure 6).

### 3.6. MSCs Decreased IL-6 Levels Independently of the Time of MSCs Administration

**Figure 7:**
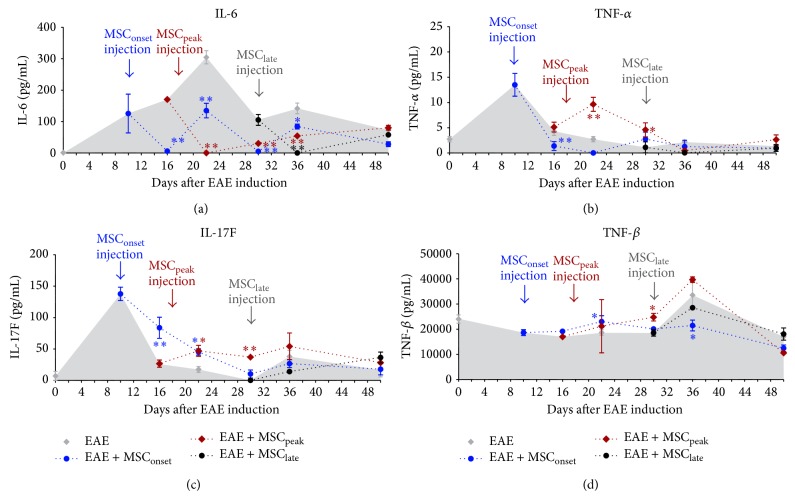
MSCs administration significantly diminished IL-6 plasma levels independently of the time of injection. Plasma samples were isolated from EAE mice, treated or not (EAE) with MSCs at different time of EAE progression, at the onset (EAE + MSC_onset_), at the peak (EAE + MSC_peak_), and at the time of EAE stabilization (EAE + MSC_late_). Blood samples were taken (day 0), at the onset (day +10), before the peak (day +16), after the peak (day +22), at the time of EAE stabilization (day +30), after EAE stabilization (day +36), and at the end of experiment (day +50). Plasma samples were isolated by centrifugation and stored at −80°C until being used. Cytokine levels IL-6 (a), TNF-*α* (b), IL-17F (c), and TNF-*β* (d) were detected using Milliplex mouse Th17 magnetic bead panel on Luminex 200 (Merck, Millipore). The samples of each EAE group (*n* = 7) were pooled and analyzed in triplicate. Bars represent the mean ± SEM; statistical differences were calculated using Mann-Whitney test (^*^ *P* < 0.05).

In order to evaluate the effect of MSCs injections on cytokine plasma levels in EAE mice, we used a Milliplex mouse magnetic bead panel kit to measure Th1, Th17, and anti-inflammatory cytokines as previously described. We observed that MSCs, independently of the time of injection, significantly decreased IL-6 levels and this effect was maintained over time (^*^ *P* < 0.005, ^**^ *P* < 0.005 as indicated in Figure 7(a)). MSCs injected at the onset of EAE were also able to decrease the levels of TNF-*α* (^**^ *P* < 0.005) (Figure 7(b)). We also observed an increase of IL-17F (^**^ *P* < 0.005) (Figure 7(c)) and TNF-*β* (^*^ *P* < 0.05) (Figure 7(d)), without a change in IL-17A levels (Supplementary Figure 3). MSCs injected at the peak of EAE also induced an increase in IL-17F and TNF-*β* levels (^**^ *P* < 0.005, ^*^ *P* < 0.05, resp.) (Figures 7(c) and 7(d)), accompanied also with an increase of TNF-*α* (^*^ *P* < 0.05) (Figure 7(b)). IL-17A levels were below the limit of detection in this case. Late administration of MSCs in EAE mice, at the time of EAE stabilization, had not a significant effect in any of the cytokines evaluated. However, it is important to mention that cytokine levels were extremely low at this point. Finally, very low levels of IL-4, IL-23, and IFN-*γ* were detected in all EAE groups after the onset of EAE, and no significant changes were observed after MSC administration (Supplementary Figure 3). We did not detect significant plasma levels of IL-10 and IL-1*β* in any group (data not shown).

## 4. Discussion

In this study, the therapeutic effects of MSCs administration at different times of EAE progression, associated with different inflammatory states of the disease, were evaluated. Unexpectedly, the appearance of atypical signs of EAE was observed when MSCs were injected after the onset of EAE. Atypical EAE is characterized by ataxia and an immune response biased to Th17 phenotype, in striking contrast to classical EAE, characterized by ascending paralysis and Th1 mediated immune responses. The effects of MSCs treatment at different time points during EAE were analyzed to bring awareness of how important is the currently inflammatory condition of the patient, at the moment of MSCs administration as cellular therapy to MS patients.

It was observed that injections of MSCs at early stages of EAE progression (EAE + MSC_onset_ or EAE + MSC_peak_ groups) induced an improvement of daily classical clinical score and also a reduction of the AUC, but no beneficial effect was observed when MSCs were injected at the time of EAE stabilization (EAE + MSC_late_) (Figure 3). These results correlate with higher levels of proinflammatory cytokines in EAE mice at the time of MSCs administration (Figure 6). These results are in agreement with previously published data showing that early administration of MSCs reduced the disease severity, but no effect was observed when MSCs were injected later [14].

The appearance of atypical signs in EAE mice after MSCs injection is an intriguing and novel finding. It was observed that MSCs significantly induced the appearance of atypical signs of EAE when injected at the peak and at the time of EAE stabilization, but not during early time points (Figure 4). It is presumed that these atypical signs are due to a predominance of Th17 lymphocytes within the CNS [28, 29], contrary to classic EAE, which is governed by Th1 lymphocytes [25]. Therefore, the presence of Th1, Th17, and also Treg lymphocytes was evaluated within the CNS after MSCs injections. We observed, independently of the time of administration, that MSCs are able to diminish the expression of T-bet, ROR*γ*T, and Foxp3, suggesting a lesser presence of Th1, Th17, and Treg, respectively, in brain samples (Figure 5). However, decreases of brain proinflammatory lymphocytes were not always associated with an attenuation of the clinical score, especially in EAE + MSC_late_ (Figure 3(c)). We speculate that as the damage caused by demyelination in the CNS occurs early, therefore the symptoms might be reversed only by MSCs acting at early time points. Later administration of MSCs, despite decreasing the presence of T cells on EAE-brain, was not efficient for improving clinical signs of EAE.

Although late administration of MSCs induces atypical signs of EAE, no association was found with ROR*γ*T/T-bet ratio, as previously described by Stromnes et al. These authors evaluated Th1/Th17 ratio by ELISPOT assays using lymphocytes derived of spleen and CNS samples obtained at the peak of EAE [28]. Furthermore, there was no increase of ROR*γ*t in brain samples (Figure 5(b)) or percentage of CD4^+^IL17^+^ cells in lymph nodes as previously described [32], probably because the T cell analysis was performed fifty days after EAE induction in order to detect late changes in the clinical score. It is expected that, at day 50 after EAE induction, brain inflammation was already resolved and lymphocyte levels are much lower to find differences in Th17 and Th1.

The highest Foxp3/ROR*γ*T and Foxp3/T-bet ratios were observed in brain samples of EAE mice treated at the onset, suggesting a predominance of Treg over Th1 and Th17. It is possible that the lowest clinical score observed in EAE + MSC_onset_ animals may be due to the higher presence of Foxp3 expression (Figure 3(a)). On the contrary, EAE + MSC_peak_ and EAE + MSC_late_ showed Foxp3/ROR*γ*T and Foxp3/T-bet ratios <1. These experimental groups also showed a higher incidence of atypical signs. This result suggests that MSCs injection in the most chronic inflammatory microenvironments (i.e., the peak and the stabilization of the disease) may generate an imbalance between Treg cells and inflammatory T cells, Th1, and Th17 and possibly generate a phenotype defined as atypical EAE. Further experiments including* in situ* FACS are needed to confirm this.

Previously we described that MSC injected at the peak of EAE significantly reduced the percentage of Th17 cells, increased the percentage of Th1 cells, and increased the percentage of Treg cells on lymph nodes [32]. Now, different results were found in brain samples. Whether this is due to the tissue where the lymphocyte populations are being measured (lymph nodes versus brain tissue) or the method of analysis (flow cytometry versus transcription factor mRNA) was discarded. We performed* in vitro* differentiation assays using C57BL/6 naïve CD4+ T cells using specific cytokines (see Supplementary Methods) and determined the percentage of CD4^+^IFN*γ* ^+^ (Th1), CD4^+^IL17^+^ (Th17), and CD4^+^Foxp3^+^ (Treg) lymphocytes by flow cytometry, as well as the relative expression of T-bet, ROR*γ*T, and FoxP3 by real time PCR in sister cultures for each polarizing condition. A positive correlation between mRNA levels and the amount of cells was observed for specific T subsets measurements using these two techniques (Supplementary Figures 1a and 1b). Increased T-bet mRNA levels were detected where increased percentage of CD4^+^IFN*γ* ^+^ lymphocytes was measured by FACS, indicating that T-bet mRNA levels could be used as an alternate parameter for Th1 determination in a sample containing mixed populations of T cells. Similar results were obtained for Th17 (ROR*γ*T versus CD4+IL17+) and Treg (Foxp3 versus CD4^+^Foxp3^+^, Supplementary Figure 1).

IL-6 is a pleiotropic cytokine with important functions on different components of the immune system and is essential for Th17 differentiation [33]. IL-6 combined with TGF-*β* is necessary to induce the activation of ROR*γ*t and Th17 differentiation [34], whereas TGF-*β* by itself induces Treg differentiation. Moreover, IL-6 is able to inhibit TGF-*β*-induced Treg. Thus, the differentiation process of both cell types of cells is closely related [35, 36]. An imbalance of Treg/Th17 is an important factor for triggering an autoimmune disease. We observed that MSCs injection in EAE mice, regardless of the time of administration, dramatically decreases IL-6 which should promote the differentiation of Treg* in vivo*. Previous results from our laboratory showed that early injection of MSCs was able to induce a Treg response [32], which could explain the reduction of clinical score. Additionally, we observed a higher proportion of Treg (Foxp3/ROR*γ*T ratio > 1) in EAE + MSC_onset_, where there was an early and prolonged decrease in circulating IL-6, providing additional evidence that MSCs-induced drop in IL-6 levels might promote Treg and decrease EAE. Furthermore, it has been described that IL-6 has important effect on the CNS and has been implicated in different diseases such as MS, Parkinson, and depression [37]. Also, it has been demonstrated that IL-6 increased the permeability of the BBB [38–40]. BBB consists of network of endothelial cells closely linked by tight junctions, forming a barrier with low permeability; therefore, the infiltration of lymphocytes into CNS is limited. The breakdown of BBB facilitates the infiltration of immune cells into the brain. Taken together, we propose that the inhibition of IL-6 allowed reestablishing, to some degree, the well-functioning of the BBB; as a consequence, we observed a decreased number of infiltrating leukocytes across the BBB cell layer, despite the persisting differentiation and activation occurring in the lymph node or spleen as previously described.

Previous studies from our laboratory show that late additions of MSCs to CD4^+^ grown in polarized conditions towards Th1 or Th17 lineages inhibited Th1, but not Th17. This leads to a paradoxical increase of Th17 cells in specific MSCs-mediated immunosuppressive conditions [20]. Guo et al. also reported similar results. Fetal bone marrow-derived MSCs added to CD4^+^ T cells (MSCs/CD4^+^ 1 : 10 ratio) inhibit Th1 but still promote the expansion of human Th17 cells [21]. Our* in vivo* results using the EAE model demonstrated no increase in ROR*γ*T mRNA levels in the brain, and we have previously reported that there is no increase of Th17 lymphocytes in lymph nodes of EAE mice treated with MSCs [32]. Cytokine analysis of EAE treated mice did not show an important increase of Th17 associated cytokines, IL-17A and IL-23; only an effect was observed on IL-17F at early time of MSCs injection. However, we observed the appearance of atypical signs of EAE, which is described to be associated with an increase in Th17 response, at late time of MSCs injection. An important decrease of Foxp3/ROR*γ*T and Foxp3/T-bet ratios (<1) was detected in EAE + MSC_peak_ and EAE + MSC_late_ groups. Discrepancies could be explained by the timing of mRNA analysis, at the end point of experiment that could decrease sensitivity in detection of infiltrating Th17 into the brain after EAE score is decreased.

The appearance of atypical EAE when MSCs were injected after the onset of EAE surprised us greatly. In striking contrast, MSCs injected at the onset were highly effective. What are the reasons that may explain this difference? It is well known that the MSCs are “licensed”* in vitro* by proinflammatory cytokines such as IL-1*β*, IFN-*γ*, and TNF-*α* [41]. In view of our results, we favor the notion that the* in vivo* inflammatory microenvironment influences the immunosuppressive properties of MSCs when administered to EAE mice. MSCs injected at the onset of the disease effectively reduce both classic and atypical clinical scores. At the onset of EAE, cytokine levels were high. IL-6 and Th1 and Th17 associated cytokines were 10 to 100 times higher than before EAE induction. We propose that the inflammatory microenvironment that MSCs find at the onset of EAE is efficient to induce an* in vivo* licensing of MSCs, enhancing the immunosuppressive capabilities of MSCs. It has been long time proposed that MSCs need the presence of inflammatory cytokines to become immunosuppressive. Initial findings with combinations of splenic T-cells and MSCs derived from knockout to either IFN-*γ* or IFN-*γ*R indicate IFN-*γ* is essential to mediate this* licensing* process in MSCs. However, it also requires the combination of other proinflammatory cytokines to achieve a potent immunosuppressive phenotype [42, 43]. We must consider that MSCs are exposed to a number of other stimuli, both activating and inhibitory signals. The balance of these signals ultimately determines the immunosuppressive potency of MSCs. In agreement, we have recently reported that MSCs are able to express a number of immunosuppressive mediators (NO2, TGF-*β*1, Cox-2, and PGE2) after being cultured with supernatants from both Th1 and Th17. However, combination of two to three recombinant cytokines is imperative to reproduce this effect [32]. Interestingly, Luz-Crawford et al. demonstrated the important role of IL-17 as a “licenser” cytokine, which is consistent with the greater immunosuppressive capacity of MSCs injected at the onset, when the higher levels of Th17 associated cytokines (IL-17A, IL-17F, and IL-23) were observed [32]. MSCs injected at the peak or at later stages of EAE were inefficient in reducing the clinical score and even induced the appearance of atypical signs of EAE. It is possible that the lower levels of proinflammatory cytokines were insufficient to properly license the MSCs. Moreover, high levels of IL-27 at the peak of the disease were observed, which remained elevated until day 30 of EAE (Supplementary Figure 2). The IL-27 anti-inflammatory cytokine is associated with a regulatory profile [43], and the presence of this cytokine combined with a decrease in proinflammatory cytokines after the onset of EAE may be an important factor in determining the* in vivo* licensing of MSCs.

The final goal of this work is to call attention in the use of MSCs for the treatment of autoimmune diseases, especially to multiple sclerosis, which is characterized by heterogeneous clinical manifestations. MS patients show a diverse response to the treatment with immunomodulatory agents. Although MSCs have potent immunosuppressive capacity upon a licensing process* in vitro*, the administration of MSCs could induce unexpected effects. For example, in our EAE model, application of MSCs at the peak and at the time of EAE stabilization was able to induce the preferential appearance of atypical signs of the disease, probably by promoting an imbalance of Th1/Th17 versus Treg lymphocytes which could be associated with inefficient* in vivo* licensing process. Therefore, it is important to accurately assess the inflammatory status of patients prior to treatment with MSCs.

Further studies will allow us to better understand the complex process of MSC-mediated regulation of the immune system and to accurately predict how the patients will respond to MSC therapy.

## Supplementary Material

Supplementary figure 1: Correlation of Th1, Th17 and Treg analysis by flow cytometry and RT-qPCR.
