# Supplementary material for: Intravenous Administration of Bone Marrow-Derived Mesenchymal Stem Cells Induces a Switch from Classical to Atypical Symptoms in Experimental Autoimmune Encephalomyelitis
Source: Stem Cells Int. 2015 Mar 9;2015:140170. doi: 10.1155/2015/140170 (PMC4369963; doi:10.1155/2015/140170)
Supplement: Supplementary file 1 — Supplementary figure 1: Correlation of Th1, Th17 and Treg analysis by flow cytometry and RT-qPCR. [file 140170.f1.pdf]

## SUPPLEMENTARY METHODOLOGY

### Associated to Supplementary Figure 1

CD4+T cells were purified from splenocytes using Dynabeads Untouched Mouse Kit CD4+ T cell Isolation (Invitrogen) according to manufacturer's instructions.
For Th1, Th17 and Treg differentiation, CD4+T cells were cultured at $1x10^6$ cells/$cm^2$ in RPMI 1640 Glutamax, 10% FBS, 100U/ml Penicillin and 100 µg/ml Streptomycin, β-mercaptoethanol 55 µM at 37°C, 5%$CO_2$. CD4+ T cells was stimulated with 2.0 µg/ml anti-CD3 (BD Biosciences) and 1.5 µg/ml anti-CD28 (BD Biosciences). Skewing conditions for T helper differentiation were as follow: Th1 differentiation, 10 ng/ml IL-12 (R&D Systems, USA) and 2.5 µg/ml anti-IL-4 neutralizing antibodies (BD Biosciences). Th17 differentiation, 50 ng/ml IL-6 (R&D Systems), 5 ng/ml TGF-β1 (BioVision USA) and 2.5 µg/ml neutralizing anti-IFN-γ and anti-IL-4 antibodies (BD Biosciences). Treg differentiation, 10 ng/ml TGF-β1 (BioVision USA), 20 U/ml IL-2 (eBioscience) and 2.5 µg/ml neutralizing anti-IFN-γ and anti-IL-4 antibodies (BD Biosciences). Cultures were established for 5 days with 50% media change every 48 h. For FACS assays, T cells were re-estimulated with PMA/Ionomycin/Brefeldin-A for 4 h at 37°C before staining.

For RT-qPCR experiments, RNA extraction was carried out using the RNeasy mini kit QIAGEN (Biosonda) according to manufacturer´s instructions. The synthesis of cDNA was carried out using 2 µg of total RNA. PCR was performed using diluted cDNA (1:3) and 10 µl of primer-containing GoTaq MasterMix (Promega, 300 pmol each primer) and analyzed using Mx3000P qPCR system (Agilent Technologies). Primers used and protocol for expression levels determination are described previously in the methodology.

## SUPPLEMENTARY FIGURES

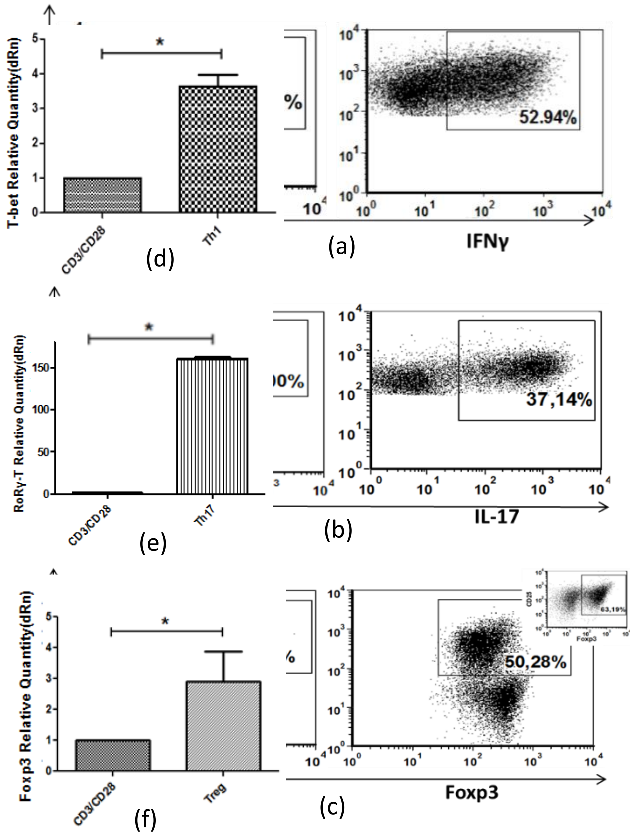


**SUPPLEMENTARY FIGURE 1:** Correlation of Th1, Th17 and Treg analysis by flow cytometry and RT-qPCR. Naïve CD4+ cells isolated from C57BL/6 spleens were differentiated into Th1, Th17 and Treg lineages using specific panel of cytokines (see supplementary methods). After 5 days *in vitro*, cells were stained for CD4 and either IFNγ, IL-17 or FoxP3. Flow cytometry analysis of Th1 (CD4+IFNγ+, (a), Th17 (CD4+IL17+, (b) and Treg (CD4+Foxp3+/CD4+CD25+Foxp3+, (c) populations are shown. Relative mRNA levels of the transcription factors T-bet (d), RORγT (e) and Foxp3 (f) were determined by RT-qPCR from experiments performed in parallel to the FACS assays. A positive correlation between T-bet, RORγT and Foxp3 mRNA levels and the percentage of Th1, Th17 and Treg cells was respectively detected in our differentiation assays.

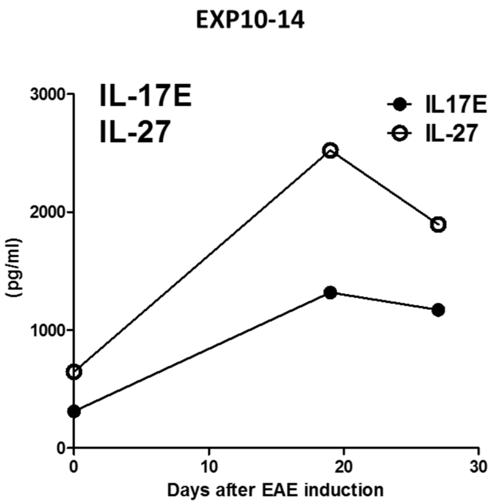


(a)

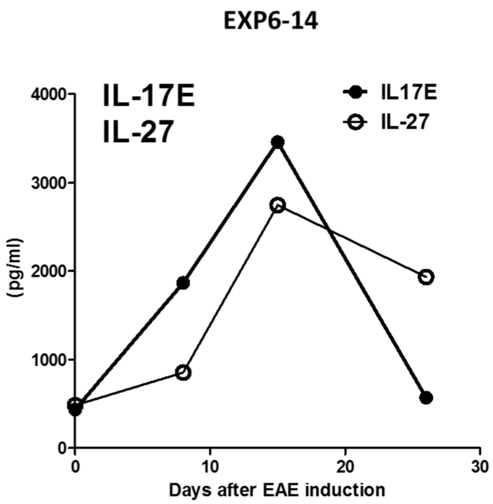


(b)

**SUPPLEMENTARY FIGURE 2: IL-27 and IL17F plasma levels at different times of EAE progression.** Blood samples from affected animals from two independent EAE experiments were taken at indicated times of EAE progression and pooled (n=7). Plasma samples were isolated by centrifugation and stored at -80°C until use. Cytokine levels of IL-27 and IL-17E were detected in triplicate using Milliplex mouse Th17 magnetic bead panel on Luminex 200TM (Merck, Millipore). a) EXP10-14 and (b) EXP6-14.

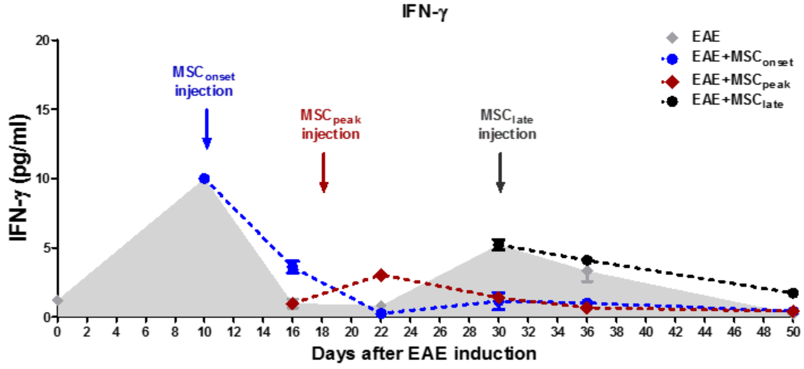


(a)

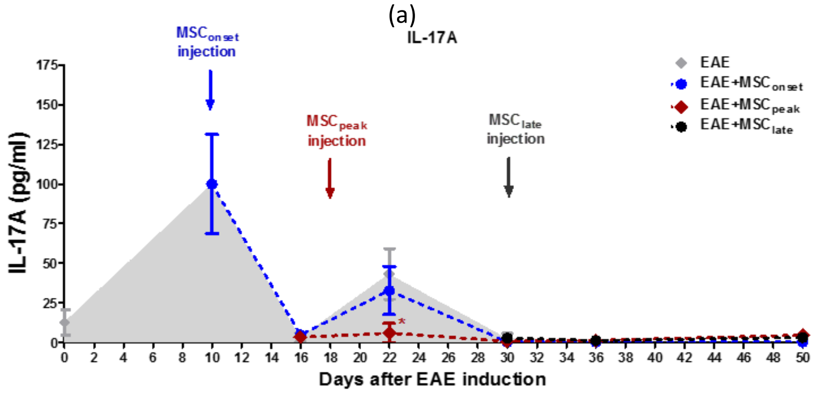


(b)

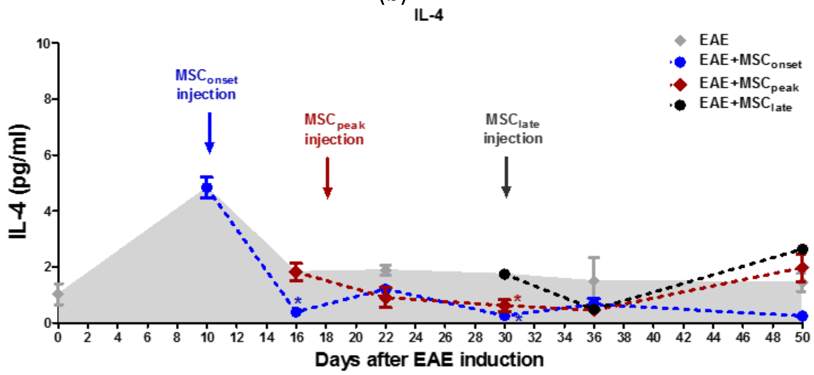


(c)

**SUPPLEMENTARY FIGURE 3: Cytokine levels after MSCs injections at different times of EAE progression.**

C57BL/6 mice were immunized with $MOG_{35-55}$ to induce EAE and treated with MSCs at different time points of disease progression, i.e. at the onset (EAE+MSConset), at the peak (EAE+MSCpeak), and at the time of EAE stabilization (EAE+MSClate). Untreated EAE mice received vehicle (PBS) only on day 10. For all animals, blood samples were taken before EAE induction (day 0), at the onset of EAE (day +10), two days before the peak (day +16), after de peak (day +22), at the time of EAE stabilization (day +30), after EAE stabilization (day +36) and at the end of experiment (day +50). Blood samples were pooled (n=7) and plasma samples were isolated by centrifugation and stored at -80°C until used. Cytokine levels of INF-$\gamma$ (a), IL-17A (b) and IL-4 (c) were determined using Milliplex mouse Th17 magnetic bead panel on Luminex 200TM (Merck, Millipore). The samples of each EAE group were

pooled and analyzed in triplicate. Bars represent the mean ± SEM, statistical differences were calculated using Mann–Whitney test (*, $p<0.05$).
